# Humeral lengthening and deformity correction

**DOI:** 10.1007/s11832-016-0789-6

**Published:** 2016-11-08

**Authors:** Gamal Ahmed Hosny

**Affiliations:** Orthopaedic Department, Benha University Hospitals, 11 Al Israa Al-mohandeseen Street, Cairo, Egypt

**Keywords:** Humeral lengthening, Deformity, Ilizarov principles

## Abstract

**Introduction:**

Ilizarov principles and hybrid fixation have improved the results of humeral lengthening. We reviewed the literature on humeral lengthening using different fixators with regard to indications, operative technique, results and complications. We also retrospectively reviewed 56 segments in 46 patients treated with humeral lengthening and deformity correction using Ilizarov external fixation. The etiology was achondroplasia (10 patients), epiphyseal injury (8 cases), infection (11 cases) and Erb’s palsy (17 cases). The average age at surgery was 14 years (range 8–20 years). The patients were assessed clinically and radiographically and DASH score was available for 36 segments. Follow-up ranged from 1−11 years. The magnitude of lengthening achieved ranged from 5−15.5 cm with an average of 9 cm. The average healing index was 29.5 cm (range 26–37 days). The percentage of area of lengthening to the original length ranged from 25 to 100% with an average of 55%. The average DASH (available for 36 segments only) score ranged from 15−40 preoperatively to 7−16 (*P* = 0.04) at last follow-up. Functionally, all the patients returned to their preoperative jobs and daily activities including sports.

**Complications:**

Complications included pin track infection in 46 segments, radial nerve palsy which recovered completely in 2 patients, fracture of the regenerate in 7 cases and premature consolidation of the regenerate in one case.

**Conclusion:**

Humeral lengthening, whether unilateral or bilateral, is a valid method that improves the outcome following arm shortening and deformity correction, including angulation and rotation. Extensive lengthening up to 100% of the original length could be achieved without increasing the risk of complications.

**Level of evidence:**

IV, retrospective cohort.

## Introduction

Bone lengthening and deformity management of the lower limbs is a standard technique with an increasing number of reports in the English literature. On the contrary, there are few papers regarding upper extremity lengthening. Dick and Tietjen published the first case of humeral lengthening in 1978 [1]. Previous opinions assessed the functional risk of humeral lengthening to outweigh its benefits [2], and humeral lengthening was regarded primarily as a cosmetic procedure. However, recent publications suggest that the goals of bilateral humeral lengthening in achondroplasia are not just cosmetic but to restore the proportions between upper and lower limbs, improve reach, and increase the ability to perform perineal personal hygiene [3–5]. External fixators including unilateral, multiaxial or circular have been used and recently intramedullary lengthening devices have been introduced for the same purpose [6–8]. Nevertheless, Ilizarov’s law of tension stress has been the mainstay of treatment [9]. Unilateral humeral lengthening is usually performed to correct deformities where angulation is >20 degrees or rotational and limb length inequality is >5 cm. Proximal humeral physis is responsible for approximately 80% of the humeral length. Therefore, septic epiphysitis or trauma can cause premature fusion and significant shortening [10, 11]. Premature closure of the medial part and continuation of growth of the lateral part can lead to varus angulation [12]. Angulation of <20 degrees does not result in functional or cosmetic problems and does not usually require surgical interference [13, 14]. The aim of this study was to review the literature on humeral lengthening, and delineating the indications, results and complications. We also reviewed the results of 56 operations for humeral lengthening and deformity correction, performed by a single surgeon using the circular frame.

## Methods

From 2002 until 2013, 50 cases with humeral shortening of >5 cm were referred to our center. Four cases were excluded due to inadequate follow-up data. Bone transport cases were not included in this study. Therefore, 56 lengthening procedures were retrospectively reviewed. Angular deformities of >20 degrees were evident in 28 segments (range 20–50 degrees) and internal rotation deformity in three segments (range 30–45 degrees). There was associated fixed elbow flexion deformity in four cases. There were 10 bilateral cases. Therefore, the etiology was achondroplasia (10 patients), epiphyseal injury (8 cases), infection (11 cases) and Erb’s palsy (17 cases). The average age at surgery was 14 years (range 8–24 years). The waiting period ranged from 5−10 days according to the age of the patient and the degree of soft-tissue dissection. The rate of distraction was one millimeter per day which was modified depending upon the rate of regenerate formation.

**Fig. 1:**
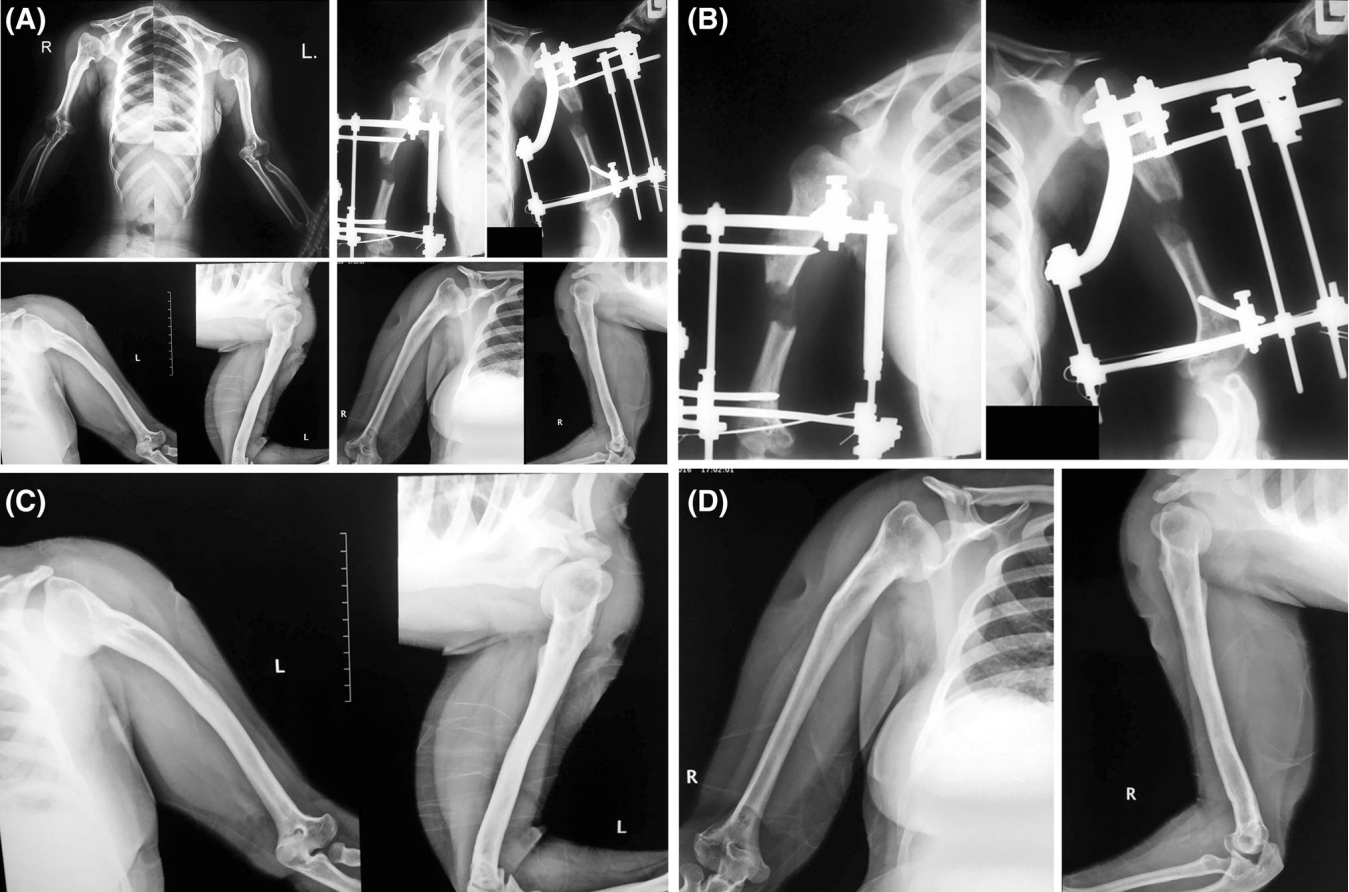
**a** Anteroposterior radiograph of both humeri in a 17-year-old girl with achondroplasia. **b** X-ray during lengthening. **c** Anteroposterior and lateral X-rays after 7-year follow-up. **d** Anteroposterior and lateral X-rays after 7-year follow-up

All the operations were performed by the author according to Ilizarov principles. The proximal part of the frame was composed of either a 90 or 120 degree arch fixed to the bone with two half pins (4.5 or 6 mm diameter according to the bone diameter) with an angle of approximately 90 degrees in between. A third half pin was applied using a rancho cube, making an angle which bisects the angle between the first two pins. Distally a 5/8 ring is applied above the olecranon fossa and mounted to the bone with two wires (1.8–2 mm) with an angle of approximately 30 degrees in between. Another half pin is applied proximally vertical to the K-wire from posterior to anterior (Fig. 1). Sometimes, an additional ring was added to the distal part of the construct with another K-wire for fixation. Osteotomy was performed in the middle third of the bone distal to the deltoid tuberosity through a small anterolateral approach. In six cases, the posterior approach and exposure of the radial nerve was performed prior to fixator application due to the difficulty of identifying anatomical landmarks on a very short humerus.

For unilateral and multiaxial frames, the cannulated wire technique can be used. The first K-wire was introduced just above the olecranon fossa perpendicular to the bone under fluoroscopic control followed by cannulated drill and a 6-mm hydroxyapatite-coated half pin. The most proximal pin is inserted in the deltoid area. The position of the proximal pins is anterior and lateral. The middle two pins are inserted in the monolateral frame which is used as a guide for insertion. In cases with deformity, the proximal and distal parts of the frame are placed perpendicular to their corresponding segments of bone. The direction of the pins is limited to the plane of unilateral frames and mandates acute correction of the deformity. On the contrary, a multiaxial frame allows application of the half pins in different planes and gradual correction of the deformity using hinges [3, 5, 7].

The patients were discharged from hospital after 24 h. The frame was removed in the outpatient clinic after complete consolidation of the regenerate or the appearance of three intact cortices in the X-rays and splinted for 6 weeks to guard against fracture. Follow-up was every week until the end of distraction, every other week until frame removal, after 1 month, and then every 3 months for 1 year followed by yearly examination.

The patients were assessed clinically and radiographically for length inequality, elbow and shoulder range of motion (ROM) and function. Shortening <3 cm was accepted at follow-up.

### Statistical analysis

Pre- and postoperative DASH scores were compared using two-tailed paired sample *t* tests. A *p* value <0.05 was considered statistically significant.

## Results

**Fig. 2:**
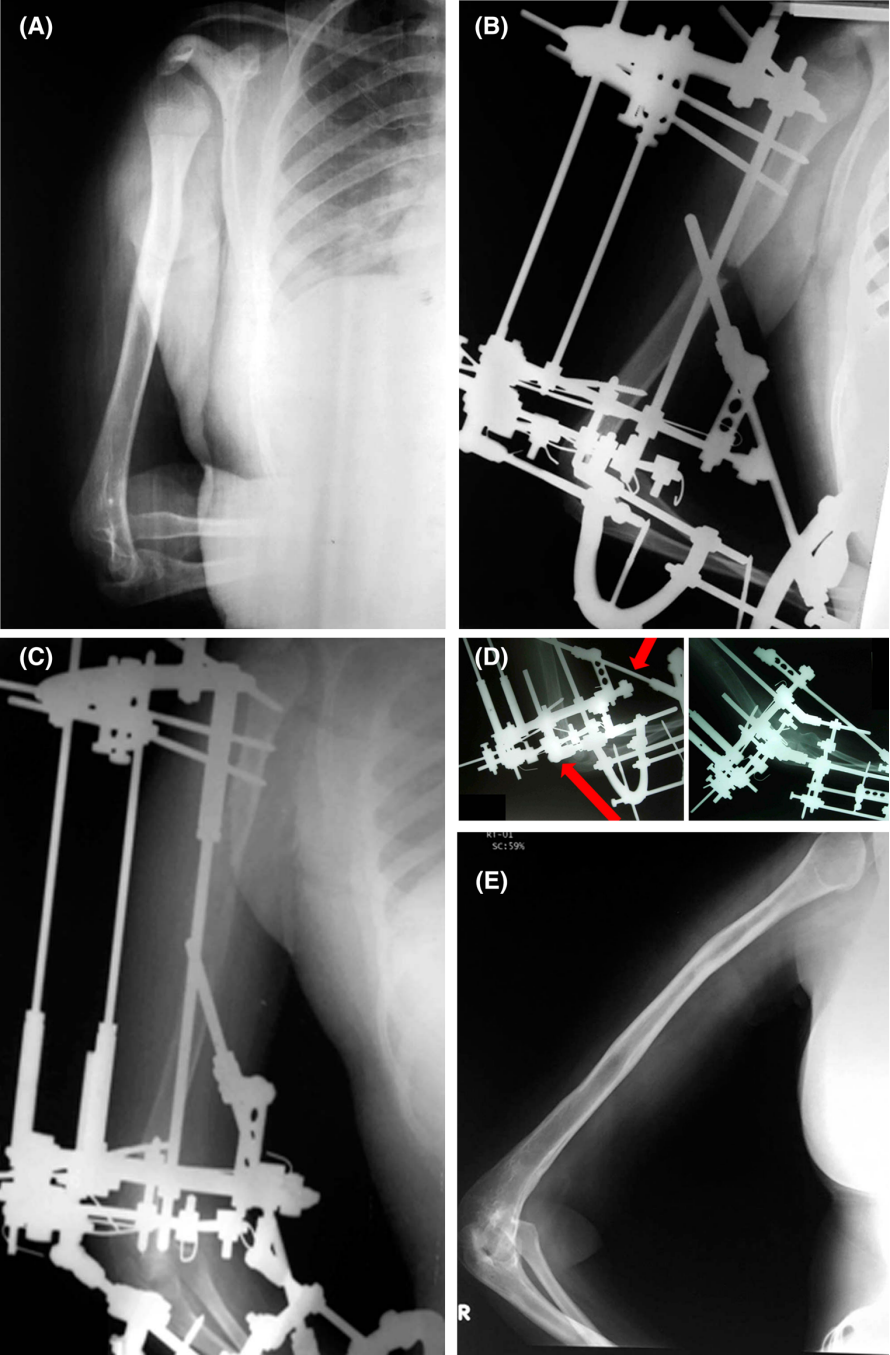
**a** A 13-year-old girl with Erb’s palsy, 9 cm humeral shortening and fixed elbow flexion (70–100 degrees). **b** X-ray after frame application to the humeri and forearm. **c** X-ray at the end of lengthening. **d** X-ray in maximum flexion and maximum extension. **e** X-ray at last follow-up 4 years after frame removal

Follow-up ranged from 1−11 years (average 4.5 years). The magnitude of lengthening achieved ranged from 5−15.5 cm with an average of 9 cm. The average healing index was 29.5 days (range 26–37 days). The percentage of area of lengthening to the original length ranged from 25−100% with an average of 55%. The planned lengthening was achieved in all cases except four cases with shortening <2 cm (accepted inequality). Deformities were corrected concomitantly with distraction. Shortening and angular deformities were corrected first followed by derotation in cases with internal rotation deformity. Residual deformities <10 degrees were evident in nine segments at last follow-up. In cases with fixed elbow flexion, the frame was extended to the forearm followed by gradual extension (Fig. 2).

The average DASH (available for 36 segments only) score ranged from 15−40 preoperatively to 7−16 (*P* = 0.04) at last follow-up. Functionally, all the patients returned to their preoperative jobs and daily activities including sports. There was temporary limitation of the shoulder and elbow ROM immediately after frame removal (10–35% of the preoperative ROM) which gradually resolved between one and six months postoperatively.

## Complications

1. There was some sort of pin track infection in 46 cases (82%) which usually responded to broad-spectrum oral antibiotics (first-generation cephalosporin) for one week and increasing the frequency of dressings. Parenteral antibiotics were required in 31 segments. The infected wire or half pin was removed in the outpatient clinic in five segments during the course of treatment.
2. Radial nerve palsy in two cases—one developed 24 h postoperatively and the other at 2 weeks after surgery. In the first case we removed the possible offending wire immediately and the nerve recovered after two and a half months. The second case was treated with removal of the possible offending wire and reducing the rate of distraction to half a millimetre per day.
3. Fracture of the regenerate developed in six cases (Fig. 3). All cases were treated with splintage for 6 weeks.

**Fig. 3:**
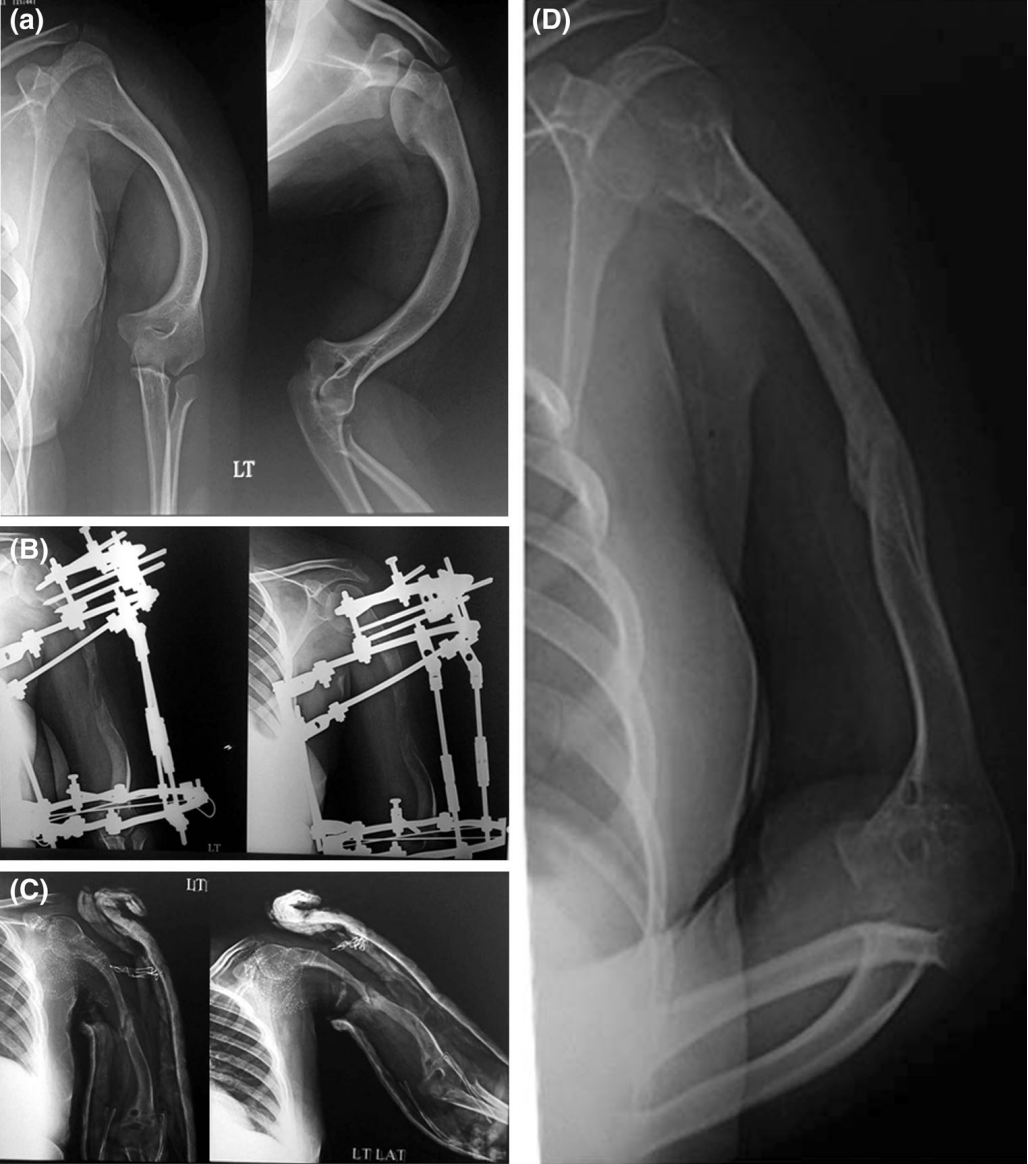
**a** A 14-year-old patient with humeral shortening and deformity. **b** X-ray at the end of lengthening and deformity correction. **c** Fracture of the regenerate after fixator removal with the arm in a splint. **d** Follow-up X-ray
4. Inferior subluxation of the shoulder developed during treatment in two cases and resolved with physiotherapy.
5. Premature consolidation of the regenerate in one case. The treatment was continuation of distraction at a rate of 1.5 mm/day which led to accumulation of the force of distraction and, finally to forcible wide separation of the osteotomy site with severe pain. The gap was then closed acutely to reduce the pain, a waiting period for 3 days followed by distraction at a rate of 1 mm/day.

## Discussion

Clear indications for humeral lengthening and deformity correction are debatable. It was thought that humeral shortening was a purely cosmetic problem [15] which may not justify the application of older methods of lengthening with a high rate of complications which may affect the upper limb function. The first reports with good results were only case reports or studies with too few patients to validate the results. The indication in bilateral cases of achondroplasia is difficult to determine as it usually follows lower limb lengthening which increases the disproportion between upper and lower limbs which can be an iatrogenic indication. Furthermore, lengthening of short stature people can be considered a cultural issue. Balci et al. [5] reported that achondroplasia patients complain about not being able to reach the perineum and perform personal hygiene independently. There was improvement of the DASH score in all patients and they were able to reach the perineal area and showed better function at preparing meals, washing hair and performing normal activities. There were many causes of unilateral humeral shortening such as upper humeral growth arrest, infection, congenital shortening and post-traumatic disorders. The usual indication for surgery in unilateral cases is shortening >5 cm and angulation >20 degrees [13, 14]. However, if lengthening is planned for this limit of shortening it is better to correct smaller degrees of deformities as well. Furthermore, if surgery is planned to correct a severe deformity, lengthening can be performed concomitantly for shorter distances from the same site as the osteotomy.

Limb lengthening procedures are fraught with complications. Increasing the area of lengthening of the lower limb usually leads to raising the risk of complications especially if it is >20% [16]. We did not consider the 20% limit of lengthening, as the percentage of the lengthening area to original length ranged from 25−100% of the original length without affecting the function. Other authors had comparable results with lengthening >20% but to shorter distances. Pawar et al. [3] reported fifteen humeri which were lengthened an average of 7 cm (range 4–9 cm), with a mean lengthening of 41% (range 23–52%). In 9 of 15 humeri for which DASH scores were available, the mean preoperative score improved from 14 to 9 after 1 year. The mean lengthening percentage was 38.4% (30–53%) in one study [17] and 60% (40–95%) in another [5]. The DASH score was determined before the operation and at last follow-up to measure physical function and symptoms of the upper limb and the presence of disabilities [18]. The mean DASH score was 32.3 (20.4–40.2) preoperatively and 9.4 (6–14.1; *p* = 0.037) at final review [5]. The gain in length was 10.2 + 1.2 cm (range 8–12 cm) after lengthening in 10 bilateral cases [19] equal to 57% (38–72%) treated by callotasis using a limb reconstruction system external fixator, with functional improvement in all cases. We had large decrease in the DASH score in our series and the range of shoulder and elbow movement was the same or better at the last follow-up in 36 segments. The functional improvement can be attributed to correction of deformities and consequently increasing shoulder joint ROM, better reach for patients with bilateral shortening and restoration of length in patients with unilateral shortening, as well as an increased ability of the patients to reach their perineum and use the bathroom independently. Therefore, humeral lengthening, even to 100% of the original length, improves upper limb function.

Many authors reported a higher rate of bone formation during distraction of the humerus [20, 21]. The healing index was 29.1 cm (range 22–35 days) after lengthening in 10 patients with achondroplasia who had no complications or sequelae [19]. Premature consolidation may be an indication of a high rate of bone formation during the lengthening process. In one study there were two premature consolidations in 24 bilateral humeral lengthening procedures [17]. The average healing index in the present series was 29.5 days/cm. We confronted this problem in only one case which we treated with continuation of distraction until the accumulated forces overcame the consolidation, leading to separation of the two ends.

Acute correction of humeral deformities at the level of the surgical neck has been reported previously [22]. Bifocal lengthening was also advised in some patients. Lengthening and deformity correction could be achieved from the same site [23]. Complex deformities of the humerus in different planes can be dealt with from a single corticotomy using a circular frame and gradual treatment. Furthermore, fixed flexion elbow joint deformities can be managed by extension of the frame to the forearm and gradual distraction.

The most common complication was pin track infection (82%). There was no consensus in the literature regarding how to manage pin track infection of the wires and half pins of the external fixators [24]. Hydroxyapatite-coated pins may reduce the rate of infection; however, we used hydroxyapatite-coated half pins in only three patients due to financial reasons.

The second most frequent complication was fracture of the regenerate (10.7%). In spite of that, however, studies revealed that the rate of callus mineralization in the humerus during distraction was higher than in the tibia and similar to that in the femur [21]. Callus formation during callotasis is probably encouraged through micromovement, as in fracture healing, where axial ‘micro motion’ is beneficial for bone regeneration and consolidation [25]. It is well known that weight-bearing is important during lower limb lengthening permitting some ‘micro motion’ across the bone gap that could enhance healing during the final phase of bone consolidation to improve the quality of the regenerate. The criteria for removal of the fixator were derived from studies of bone lengthening of the lower limb which can lead to premature extraction of the frame and a higher incidence of refracture. However, the upper limb bone is non-weight-bearing which may affect the quality of callus formation in the bone gap. Cattaneo et al. [26] reported seven fractures out of 43 humeral lengthening procedures (16%). All were treated with casting except for two that were treated with reapplication of the frame. Kashiwagi et al. [4] reported 2 fractures out of 20 lengthening procedures (10%). Therefore, we recommend protection of the regenerate with a brace for 6 weeks.

### Nerve injuries

Cattaneo et al. [26] reported one case of partial injury of the radial and ulnar nerves due to inadvertent operative distraction and two cases of radial neurapraxia during lengthening with complete recovery. Out of 20 lengthening procedures, transient radial nerve palsy developed in two cases during lengthening which was treated by stoppage of distraction [4]. Numbness persisted in one case which mandated exploration and release. The treatment was a discontinuation of lengthening and gradual compression in both cases. Ultrasonography was used in one case to show the offending agent, which was the pin in close proximity to the radial nerve as it passed along the lateral margin of the humerus [27]. There was extensive perineural scar reaction seen in this location which in part encompassed the nerve which mandated removal of that pin. Release and isolation of the radial nerve before performing the osteotomy in all cases was reported previously [28]. A posterior approach to identify the course of the nerve was performed in selected case with significant shortening to avoid iatrogenic injury during the introduction of the wires and half pins and osteotomy [29]. However, with developing experience we think this is not necessary and therefore preferred to use a small anterolateral incision in all cases except six where the arm was very short and it was impossible to identify the bony landmarks. We had only two cases (3.5%) with radial nerve palsy which developed after 24 h and 10 days postoperatively. The diagnosis was neuropraxia due to developing hematoma in the first case and nerve compression during distraction in the second case. However, we removed the wire close to the nerve in both cases. Therefore, we think the cause of the radial nerve palsy during humeral lengthening may be an immediate postoperative complication due to direct compression by a pin which needed to be removed immediately once the diagnosis was confirmed. Later, the gradual development of symptoms may be due to hematoma formation or developing perineural fibrosis close to one of the pins. Finally, during distraction, overstretch of the nerve can be the causative factor. While authors believe that stopping the lengthening procedure or even shortening has to be performed in cases of radial nerve palsy developing during lengthening, we just reduced the rate of distraction and achieved recovery of the nerve in all cases.

Inferior subluxation of the glenohumeral joint developed in four joints out of 20 segments with achondroplasia and responded well to arm sling and isometric exercises [4]. Comparison of the standing radiographs before and after the operation revealed two cases with inferior glenohumeral subluxation during lengthening (the diagnosis was achondroplasia and Erb’s palsy consequently). One of them presented with dull pain over the arm and shoulder while the other patient was symptomless. Aggressive physiotherapy was recommended daily and spontaneous reduction of the joint occurred with continuation of distraction. There was some sort of shoulder abnormality in all cases with Erb’s palsy. The involved humeral head was significantly less retroverted and in declination (medial humeral head pointed interiorly and inferiorly) relative to the noninvolved side. Osseous atrophy was present in all three dimensions and affected the entire humerus [30]. The deformity ranged from mild deformity to growth arrest of the proximal aspect of the humerus [31]. In spite of the presence of shoulder joint abnormality in all cases postoperatively, there was no deterioration of ROM at the last follow-up.

Recently, there has been a tendency to use unilateral standard frames and multiaxial frames for humeral lengthening and deformity correction to avoid the bulky circular frames and the intolerance of some patients. They are usually indicated in all cases except when complex deformities exist which mandate the use of the circular frame. After a mean follow-up of 31 months of 15 humeri in 11 patients [3], improvement of function was reported in all patients. The mean lengthening was 7 cm and the indications were growth arrest, achondroplasia and congenital short humerus. Other authors [7] reported the successful application of unilateral Wagner fixators in 11 humeri (10 patients) and achieved average lengthening of 6.2 cm with an average healing index of 32 days/cm without major complications. After an average follow-up of 40 months, Balci et al. [5] reported their results after treatment of 18 achondroplasia patients with bilateral humeral lengthening using a monorail external fixator. The mean lengthening achieved was 60% (40–90%) of the original length with increased independence of the patients at the final follow-up. The incidence and severity of complications reported with unilateral frames are similar to circular frames.

### Limitations of the study

Limitations of this study include its retrospective design, grouping unilateral and bilateral cases, and patients with different etiologies. The DASH scores were not available for all patients. However, it was important to have a reasonable number of patients to clarify many points investigated in this series regarding the safe magnitude of lengthening.

## Conclusions

Humeral lengthening, whether unilateral or bilateral, is a valid method of treatment for arm shortening and deformity correction, including angulation and rotation. Unilateral and multiaxial frames can be used efficiently in most cases. Circular fixators may be preferred in severe deformities. Extensive lengthening up to 100% of the original length could be achieved without increasing the risk of complications, regenerate formation and shoulder stability. Functional improvement is expected after surgery. There is a remarkable risk of regenerate fracture after frame removal which justifies the routine use of bracing for 6 weeks. In cases with radial nerve palsy developing during lengthening, the offending pin has to be removed immediately but there is no need to stop lengthening. Management of humeral deformities and elbow stiffness can be performed in one stage in cases with Erb’s palsy.
